# The Vocational and Educational Index: An Update to the Vocational Index to Reflect Contemporary Postsecondary Educational Options for Autistic Adults

**DOI:** 10.1007/s10803-025-06737-8

**Published:** 2025-02-22

**Authors:** Julie Lounds Taylor, Sarah Roberts Carlson, Leann Smith DaWalt, Meghan M. Burke, Grace A. Herbert, Marsha R. Mailick

**Affiliations:** 1 Department of Pediatrics, Vanderbilt University Medical Center, Nashville, TN USA; 2 Waisman Center, University of Wisconsin-Madison, Madison, WI USA; 3 Department of Special Education, Vanderbilt University, Nashville, TN USA

**Keywords:** Autism, Adult, Postsecondary education, Employment, Vocation

## Abstract

The Vocational Index, a tool to reliably capture the range of vocational and educational activities in which adults with autism engage, is regularly used in studies of adult outcomes in autism. However, recently it has been noted that there are some activities (primarily postsecondary education options) that were infrequently available when the index was developed and thus are not fully represented in the current categories. The purpose of this report is to describe the process and results of updating the Vocational Index coding categories to reflect this wider range of activities. An iterative process was used to develop updated codes (called the Vocational and Educational Index). The original Vocational Index and updated Vocational and Educational Index codes were applied to a sample of 384 autistic young adults, and differences between original and updated codes were described. The major changes to the codes involved the development of a parallel educational dimension, benchmarked to the vocational dimension in level of integration, supports, and number of hours. Applying original Vocational Index and updated Vocational and Educational Index codes resulted in few differences in the overall distribution of codes but provided additional information about the contribution of vocational versus educational activities to the overall code. Limitations of the Vocational and Educational Index and future directions for research are discussed.

**Supplementary Information:**

The online version contains supplementary material available at 10.1007/s10803-025-06737-8.

In 2012, we developed an index of vocational outcomes to capture the full range of vocational and educational activities in which adults with autism are engaged (Taylor & Seltzer, 2012). The Vocational Index addressed an emerging gap in the field as more studies began focusing on adult outcomes for autistic individuals. Though vocational and educational activities were regularly considered in studies of adulthood, they were often coded in different ways across studies, and disparate activities tended to be collapsed into large, overarching categories (e.g., sub-minimum wage work in sheltered settings and full-time competitive work in the community combined into one overarching category of “employment”). In developing the Vocational Index, we aimed to provide the research community with a way to reliably categorize and code the varied and nuanced vocational and educational experiences of adults with autism. These codes could then be used to facilitate comparisons of activities across studies, examinations of developmental trajectories throughout adulthood, and measurement of the impact of interventions and services aimed at fostering independence.

To develop the Vocational Index, we utilized employment, vocational, and educational data from nearly 350 adults with autism across six time points between 1998 and 2010 (Taylor & Seltzer, 2012). The Vocational Index consists of eleven categories coded on a nine-point scale. Coding categories encompass various combinations of vocational and educational activities, ranging from competitive employment and/or postsecondary educational (PSE) programs to no vocational or educational activities. Since its publication, the Vocational Index has become a widely utilized resource in studies involving autistic adults. It has been cited over 120 times and applied in research across various countries, including the United States (e.g., Anderson et al., 2021; Cadondon et al., 2023; Clarke et al., 2021; Lee et al., 2024; Schwartzman, 2021; Taylor & Mailick, 2014; Wehman et al., 2020), Australia (Arnold et al., 2019; Sahin et al., 2020), Japan (Otsuka et al., 2017), and Denmark (Knüppel et al., 2019). Additionally, it has been used in studies of adults with Down syndrome (Esbensen et al., 2016; Loveall et al., 2022) and fragile X syndrome (personal communication).

Since the original Vocational Index was published, there have been significant changes in PSE options for individuals with autism and other disabilities. Agencies such as the U.S. Department of Education have allocated increased funding for inclusive PSE programs for students with intellectual disability (e.g., Model Comprehensive Transition and Postsecondary Programs for Students with Intellectual Disabilities or TPSIDs) leading to a rise in such programs, as well as autism-specific support programs at colleges and universities (Grigal et al., 2018; Nachman et al., 2022). There have also been shifts to the employment landscape, some of which affect people with and without disabilities such as the rise of the gig economy, and others that are more targeted toward adults with disabilities (fueled in the U.S. by policies such as the 2014 Workforce Innovation and Opportunity Act). Given these developments, members of the broader research community (e.g., Sahin et al., 2020) have noted the need for an updated version of the Vocational Index that reflects the contemporary educational and vocational activities in which autistic adults engage. This brief report describes the process of updating the Vocational Index into the Vocational and Educational Index (VEI) and presents original and updated codes from a large sample of nearly 400 (*n* = 384) autistic adults with diverse functional abilities.

## The Process of Updating the Vocational Index

To update the Vocational Index codes, a small team consisting of a post-doctoral trainee and three Ph.D level researchers, all with extensive experience in disability and employment, reviewed over 200 interviews focused on the vocational and PSE activities of autistic young adults administered between 2020 and 2022 as a part of a larger longitudinal study. The research team examined corresponding Vocational Index codes, and identified cases where vocational and PSE activities were not captured or represented completely by the code descriptions. Examples of activities that did not fit easily into an existing code were post-high school structured internship programs and inclusive higher education programs for students with intellectual and developmental disabilities (see below for more detail).

With this information in mind, updated code descriptions were drafted by the post-doctoral trainee and revised by the Ph.D level researchers, with a focus on broadening descriptions to encompass the current variations of educational and vocational activities in which adults with autism participate. After developing the updated codes, two members of the research team independently applied the new codes to the dataset. Upon applying the codes, small adjustments were made to the coding descriptions to increase clarity, particularly in cases of disagreements between coders or when activities were not represented adequately by an existing code.

### Key Updates to the Vocational Index

Detailed descriptions of original Vocational Index coding categories and updated VEI coding categories are presented in Table 1. The most significant update involved separating out educational activities from vocational activities through the development of a parallel educational dimension, benchmarked to the vocational dimension in level of integration, supports, and number of hours. Minor changes were made to the descriptions of vocational categories to better represent the full range of contemporary vocational activities in which adults with autism engage. These changes are described below.

**Table 1:** Description of original and updated codes

Code	Original Vocational Index (from Taylor & Seltzer, 2012)	VEI	
		Vocation	Education
9	Postsecondary, degree-seeking educational program greater than 10 h a week OR employment in the community without supports greater than 10 h a week.	**Paid Community Employment without Supports**: Paid employment in a community setting with no supports above and beyond what is available to all employees. More than 10 h a week. Could be at a place of business or self-employment.	**Fully Integrated**,** Degree- or Credential-Seeking PSE Program with Typically Available Supports**: PSE program working toward a technical, associates, bachelor’s or post-bachelor’s degree, certificate, or credential. All coursework is integrated. May have access to supports through office of disability services via ADA (same as any student with a disability) or could be receiving no supports. More than 10 h of educational activities (in and outside of class) per week. Likely admitted through typical means.
8	Postsecondary, degree-seeking educational program or employment in the community without supports– total activities 10 h a week or less.	**Paid Community Employment without Supports - Minimal Hours**: Paid employment in a community setting with no supports above and beyond what is available to all employees. Ten hours or less a week. Could be at a place of business or self-employment.	**Fully Integrated**,** Degree- or Credential-Seeking PSE Program w/Typically Available Supports - Minimal Hours**: PSE program working toward a technical, associates, bachelor’s or post-bachelor’s degree, certificate, or credential. All coursework is integrated. May have access to supports through office of disability services via ADA (same as any student with a disability) or could be receiving no supports. Ten hours of educational activities (in and outside of class) or less per week. Likely admitted through typical means.
7	Employed in the community with supports greater than 10 h a week. No time spent in sheltered settings.	**Paid Community Employment with Supports**: Paid employment in a community setting with supports. Supports could be provided by a formal agency or informally (e.g., family), but must be above and beyond what employees would typically receive. More than 10 h a week.	**Fully Integrated**,** Degree- or Credential-Seeking PSE Program with Additional Disability Supports**: PSE program working toward a technical, associates, bachelor’s or post-bachelor’s degree, certificate, or credential. All coursework is integrated. PSE Program is providing additional supports as part of an autism or disability-specific program. More than 10 h of educational activities (in and outside of class) per week. Likely admitted through typical means.
6	Employed in the community with supports (no time spent in sheltered settings)– total activities 10 h a week or less.	**Paid Community Employment with Supports - Minimal Hours**: Paid employment in a community setting with supports. Supports could be provided by a formal agency or informally (e.g., family), but must be above and beyond what employees would typically receive. Ten hours or less a week.	**Fully Integrated**,** Degree or Credential-Seeking PSE Program with Additional Disability Supports - Minimal Hours**: PSE program working toward a technical, associates, bachelor’s or post-bachelor’s degree, certificate, or credential. All coursework is integrated. PSE Program is providing additional supports as part of an autism or disability-specific program. Ten hours of educational activities (in and outside of class) or less per week. Likely admitted through typical means.
5	Sheltered vocational setting and supported community employment– total activities greater than 10 h a week.	**Hybrid Vocational Activities**: Combination of segregated and integrated community vocational activities (as part of the same overall program), with payment for community employment. More than 10 h a week of all activities. OR **Unpaid internship**: A structured unpaid internship^a^ that occurs in a community setting. More than 10 h a week.	**Hybrid PSE Program**: Disability-specific PSE program that provides some segregated coursework and some coursework that is integrated with the general college population. Does not result in a degree but may receive a certificate. More than 10 h of educational activities (in and outside of class) per week.
4	Sheltered vocational setting (e.g., workshop or day activity center) with no community employment/volunteering - greater than 10 h a week OR Sheltered vocational setting and volunteering in the community– total activities greater than 10 h a week.	**Sheltered Vocational Activities**: All vocational activities are happening in segregated settings. Vocational training programs would fit here if there was no community component or if community work was unpaid. Volunteering as part of a day program would fit here. More than 10 h a week.	**Sheltered PSE Program**: Disability-specific PSE program that provides all coursework in a segregated setting (i.e., only with other students with disabilities). Does not result in a degree. More than 10 h of educational activities (in and outside of class) per week.
3	Sheltered vocational setting– total activities 10 h a week or less.	**Sheltered Vocational Activities - Minimal Hours**: All vocational activities are happening in segregated settings. Vocational training programs would fit here if there was no community component or if community work was unpaid. Volunteering as part of a day program would fit here. Ten hours or less a week.	**Sheltered PSE Program - Minimal Hours**: Disability-specific PSE program that provides all coursework in a segregated setting (i.e., only with other students with disabilities). Does not result in a degree. Ten hours of educational activities (in and outside of class) or less per week.
2	Volunteering with no other activities or postsecondary non-degree seeking education with no other activities.	**Volunteering**: Volunteer or unpaid work activities, not part of any defined program	**Non-degree seeking PSE - Minimal Hours**: Taking a PSE class or two, but not in a specific program or working toward a degree/certificate/credential.
1	No vocational/educational activities.	**No Vocational Activities**	**No Educational Activities**

Note. ADA = Americans with Disabilities Act

^a^ According to the Workforce Innovation and Opportunities Act, an internship is defined as “a planned, structured, learning experience that takes place in a workplace for a limited period of time.” (found at https://www.federalregister.gov/documents/2016/08/19/2016-15975/workforce-innovation-and-opportunity-act#sectno-reference-680.180)

### Developing Education Codes

In the original Vocational Index, descriptions of both vocational and educational activities were included within a single code. Throughout the iterative process of updating the vocational index, we identified the need to consider vocational and PSE activities separately. Thus, parallel codes were developed for vocational and educational dimensions. By implementing parallel codes and scoring these dimensions separately, different types of PSE programs are considered more fully, with an emphasis on understanding the degree of support and inclusion each program provides.

Developing parallel codes also allowed us to ensure that vocational and PSE activities with analogous levels of integration, support, and hours would receive the same code across dimensions. More specifically, the following aspects of activities were coded in similar ways across vocational and educational dimensions: (a) whether the activity occurred in a fully integrated, semi-integrated (i.e., hybrid) or fully segregated setting; (b) whether the adult received supports above and beyond what might be available to the general public; and (c) whether the activities were occurring for a minimal or greater than minimal amount of time (see Table 1). As an example, in the updated codes, supported employment in the community receives the same code as a fully integrated PSE program with disability-specific supports. In both contexts, the activity is fully integrated into the community and the adult is receiving support above and beyond what would be available to the general public. As another example of equivalence across dimensions, vocational activities in sheltered settings receive the same code as PSE programs for students with intellectual/developmental disabilities; in both cases, these programs (and the resulting code; see Table 1) are differentiated by whether they include some integrated activities (i.e., work activities in the community or classes with the general college population) or whether all activities occur in segregated settings.

Depending on the research question of interest, studies can utilize the vocational codes only, the educational codes only, or the higher of the vocational/educational codes as an overall code of vocational/educational activities. For instance, an adult who is working full-time in the community without supports (with no educational activities) would get a “9” for their vocational dimension code, a “1” for their educational dimension code and a “9” for their overall code. An adult who is in a fully integrated PSE program without supports and is not working would get a “1” for their vocational dimension code, a “9” for their educational dimension code, and a “9” for their overall code.

### Updates to Vocational Codes

In the development of the original Vocational Index, researchers carefully considered and analyzed the nature of volunteering activities, including whether such activities were a gateway to paid employment. At that time, volunteering rarely provided specific experiences or skills that led to paid work. Today, structured unpaid internships for individuals with disabilities are more common and can sometimes lead to paid employment (e.g., Project Search, Bridges; Avellone et al., 2023). Thus, in the updated VEI, codes differentiate volunteering/unpaid work from structured internships.

Another update involved clarifications around support. Throughout the process of updating the Vocational Index, we observed that participants frequently mentioned supports, trainings, and accommodations that were widely available and not offered exclusively as part of autism or disability-specific programming (e.g., a peer mentoring program at the workplace that is available to all employees; help from the writing center of a college or university available to all students). In the original Vocational Index, codes described supports as either received (i.e., supported employment) or not received (i.e., competitive employment), and it was challenging to discern how to code these more widely available supports. Thus, codes in the updated VEI give additional guidance about coding different types of job/PSE supports. Specifically, to be considered “supported,” the job or PSE program should involve supports above and beyond what would be available to other employees or students, often offered by a disability-specific program.

### Applying the Updated Coding Conventions

Administration procedures for gathering vocational and educational information used to generate VEI codes, example questions used to gather this information, and additional guidance for applying VEI codes are presented in the supplemental materials.

### Participants

To examine consistency and change between the original Vocational Index codes and updated VEI Codes, the codes were applied to a sample of 384 autistic adults. Adults were drawn from four studies of autistic adolescents and adults. Inclusion criteria and recruitment methods for each subsample are presented in the supplemental materials. Participants from each subsample were eligible for this study if they were 18 years of age or older and had exited high school (and thus had postsecondary vocational/educational information). Some adults participated in more than one study; in those cases, data from the most recent study were used. Thus, all autistic adults included in this analysis were unique, only being represented in one subsample. Further, any adults who were part of the sample used to develop the original Vocational Index (Taylor & Seltzer, 2012) were removed from this analysis.

Demographic characteristics for each subsample are presented in Table 2. Subsample 1 included participants (*n* = 59) from a randomized controlled trial of a parent advocacy intervention for youth with autism and included youth with a wide range of functional abilities (Taylor et al., 2023). Information on youth’s vocational and PSE activities was collected at multiple timepoints via interviews with the caregiver (who was the primary respondent); information collected at baseline (prior to intervention) was used in the present analysis. Subsample 2 (*n* = 82) was drawn from a study of parents of autistic adolescents and adults with low IQ (i.e., IQ scores ≤ 70). Information on youth’s vocational and PSE activities was collected via parental survey (Taylor et al., 2024). Subsample 3 (*n* = 77) was drawn from a study of autistic adolescents and adults who did not have intellectual disability and could self-report (Libster et al., in press). Vocational and PSE information was collected via interviews with the adults. Subsample 4 (*n* = 166) was drawn from a longitudinal study of autistic adults who could self-report (Mueller et al., under review). Information on the adult’s vocational and PSE activities was collected via interviews with the adults, with informants often providing additional details and context that aided in coding decisions. All the subsamples had a maximum age of 26 years, except for Subsample 4 in which adults could be up to age 35. In total, data from 384 autistic adults were included. Note that Subsample 1 was part of the sample that was used to develop the updated codes; all other subsamples had codes applied after the updating was complete.

**Table 2:** Demographic characteristics of each Subsample included in analyses

Characteristics	% (*n*) or Mean (SD)			
	Subsample 1 (*n* = 59)	Subsample 2 (*n* = 82)	Subsample 3 (*n* = 77)	Subsample 4 (*n* = 166)
Autistic adult characteristics				
Age				
Mean age	22.23 (2.06)	23.79 (1.61)	21.03 (2.06)	25.87 (4.14)
Age range	19.0–26.6	20.1–26.1	18.0–26.0	18.6–35.5
Intellectual Disability/Low IQ				
Intellectual disability/IQ 70 or below	33.9% (20)	100% (82)	0	7.8% (13)
No intellectual disability/IQ above 70	66.1% (39)	0	100% (87)	92.2% (153)
Gender				
Male	71.2% (42)	78.0% (64)	70.1% (54)	60.2% (100)
Female	25.4% (15)	22.0% (18)	19.5% (15)	26.5% (44)
Non-binary gender or other	1.7% (1)	0	2.6% (2)	6.6% (11)
Did not report	1.7% (1)	0	7.8% (6)	6.6% (11)
Race				
White	79.7% (47)	81.7% (67)	76.6% (59)	85.5% (142)
American Indian	0	0	1.3% (1)	0
Asian	1.7% (1)	3.7% (3)	2.6% (2)	1.2% (2)
Black or African American	8.5% (5)	8.5% (7)	0	4.8% (8)
Native Hawaiian or Pacific Islander	0	1.2% (1)	0	0.6% (1)
Other	1.7% (1)	0	1.3% (1)	3.0% (5)
More than one Race	8.5% (5)	4.9% (4)	10.4% (8)	4.2% (7)
Did not report	0	0	7.8% (6)	0.6% (1)
Ethnicity				
Hispanic/Latino	8.5% (5)	8.5% (7)	- - -	5.4% (9)
Not Hispanic/Latino	91.5% (54)	91.5% (75)	- - -	93.4% (155)
Did not report	0	0	100% (77)	1.2% (2)
Living situation				
Living with parent	88.1% (52)	86.6% (71)	85.7% (66)	56.6% (94)
Not living with parent	11.9% (7)	13.4% (11)	14.3% (11)	43.4% (72)
Highest degree completed				
High school diploma or less	- - -	100% (82)	84.4% (65)	65.1% (108)
Associates or technical degree	- - -	0	3.9% (3)	10.2% (17)
Bachelor’s degree	- - -	0	2.6% (2)	21.1% (35)
Graduate or professional degree	- - -	0	0	3.6% (6)
Did not report or “other” without specification	- - -		9.1% (7)	0
Informant characteristics (only for studies where primary work/education information was collected from informants)				
Mean age	53.21 (6.15)	54.75 (6.36)	- - -	- - -
Relationship to adult				
Mother (biological, step, adoptive)	86.4% (51)	93.9% (77)	- - -	- - -
Father (biological, step, adoptive)	13.6% (8)	4.9% (4)	- - -	- - -
Other	0	1.2% (1)	- - -	- - -
Highest level of completed education				
High school diploma or less	8.5% (5)	13.4% (11)	- - -	- - -
Some college but no college degree	16.9% (10)	13.4% (11)	- - -	- - -
Associates or technical degree	8.5% (5)	15.9% (13)	- - -	- - -
Bachelor’s degree	40.7% (24)	32.9% (27)	- - -	- - -
Master’s degree	16.9% (10)	18.3% (15)	- - -	- - -
PhD or professional degree	8.5% (5)	6.1% (5)	- - -	- - -

Note. Completed education (beyond high school) for the autistic adult was not asked consistently for Subsample 1 and ethnicity data was not gathered consistently for Subsample 3. Subsamples 1 and 4 had comprehensive data to make determinations about whether the autistic adult had intellectual disability or not. Subsamples 2 and 3 had standardized IQ information (which was used as recruitment criteria) but not standardized adaptive behavior nor information about intellectual disability diagnosis

### Procedure for Applying Codes

We used the following procedures for applying codes: (1) a member of the research team (typically paid research assistants) extracted the data from the interviews/questionnaires necessary to determine the code; (2) the research team member that extracted the information then applied the original and updated codes to the vocational and PSE activities of each sample member; (3) senior members of the research team (i.e., the first author or another senior team member with expertise in autism and employment) applied the codes to those same sample members, noting cases where there was disagreement in codes; (4) the two researchers who applied codes used a case consensus process to resolve disagreements, which sometimes involved revisiting the data to search for additional details that were not included in the initial data extraction; and (5) any remaining disagreements were discussed with the full research team for that study, and were resolved based on that discussion. To establish inter-rater reliability, a third, independent research team member applied the Vocational Index and VEI codes to a random 20% of cases (*n* = 82). The percent agreement was: 92.7% for original Vocational Index codes, 93.9% for VEI vocational dimension codes, 97.6% for VEI educational dimension codes, and 95.1% for VEI overall codes.

### Distribution of Original and Updated Codes

The percentage of the full sample who received each of the original Vocational Index and updated VEI codes are presented in Table 3. Original and updated codes for each subsample are presented in the supplemental materials. As can be seen in Table 3, just under 50% of the full sample had an overall code of “9” indicating participation in competitive employment or participation in a degree or certificate-seeking PSE program (without autism-specific supports) for over 10 h a week. There was very little change in the percentage of the sample who received a code of “9” from the original to the updated overall codes. Adults who received a “9” in the original code, but not in the updated overall code, were those who had been admitted to college via typical avenues and received more extensive supports than available to the general student population via an autism-specific support program. These supports were not considered in the original codes but are coded similarly to supported employment in the community in the updated codes.

**Table 3:** Distribution of original and updated Vocational Index codes

	Percent (*n*) of sample with each code				
Code	Original Vocational Index		VEI		
			Overall	Vocation	Education
9	45.6% (175)		45.3% (174)	31.5% (121)	20.3% (78)
8	6.8% (26)		7.0% (27)	6.8% (26)	4.7% (18)
7	5.2% (20)		6.0% (23)	5.7% (22)	1.0% (4)
6	2.3% (9)		2.6% (10)	2.9% (11)	0
5	1.8% (7)		2.3% (9)	2.1% (8)	1.3% (5)
4	8.6% (33)		9.1% (35)	8.3% (32)	1.3% (5)
3	3.1% (12)		3.1% (12)	3.1% (12)	0
2	5.7% (22)		3.6% (14)	6.0% (23)	2.3% (9)
1	20.8% (80)		20.8% (80)	33.6% (129)	69.0% (265)

Note. Overall Code is the higher of the VEI vocational or educational code for each person

Approximately 25% of the sample received a code of “2” or “1” both in the original and updated overall codes, indicating no activities, volunteering only, or PSE that was not working toward a degree or certificate. The largest difference between the original Vocational Index codes and the overall VEI codes was in a code of “2,” with the percentage of the sample with that code dropping from 5.7% in the original code to 3.6% in the updated overall code. There were three phenomena that accounted for this difference: (1) unpaid vocational activities as part of a defined internship program were given a code of “2” (i.e., volunteering) in the original codes and a code of “5” (i.e., structured internship) in the updated codes; (2) PSE programs for students with intellectual disability that are certificate- (but not degree-) seeking were given a code of “2” in the original codes, but are considered a “4” or “5” in the updated codes depending on whether all coursework is specific to students with disabilities or some is integrated with the broader student body; and (3) clarifications in the updated category descriptions about how to code PSE programs that result in technical or other certifications but not degrees (see Table 1).

Relative to the original codes, the updated codes provided additional information about the contribution of vocational versus PSE activities in determining the overall code. For example, as can be seen in Table 3, a code of “9” was more often due to vocational activities than PSE activities. The ability to distinguish between dimensions also provided additional information about the types of activities in which sample members were *not* participating. Though just over 20% had no vocational nor educational activities, the numbers in the “no activity” categories were substantially higher when examining the dimensions, with one-third of adults not participating in vocational activities and about 70% of adults not participating in PSE programs (see Table 3).

### Comment

#### Contribution of Updated Codes

With the proliferation of research and programs aimed at improving employment and educational outcomes for individuals with autism as well as those with other developmental disabilities, it is important to have a rigorous and reproducible way to code these activities and capture the nuances between the activities. Reproducible coding schemes allow for the comparison of vocational activities across samples, cohorts, and countries, as well as allow for the investigation of changes in types of vocational or educational participation over time. The original Vocational Index was useful in achieving this goal (Taylor & Seltzer, 2012), but did not always capture the range and variety of PSE and vocational activities that are currently available to autistic adults (particularly those not available when the codes were developed over 10 years ago). Thus, the purpose of this study was to describe an update of the Vocational Index codes to reflect a broader range of activities that better represents the variability of contemporary activities in which adults participate, while also keeping the main distinctions between activities described in the original Vocational Index.

The most significant update to the original Vocational Index was the development of codes that capture the range of PSE activities available to autistic adults, benchmarked to the vocational domain in terms of level of integration and support. The number of PSE programs for students with intellectual disability has more than doubled since 2009 (from 148 to 333; Grigal et al., 2018; Think College.net) and the number of college support programs specifically for autistic students has more than quadrupled since 2016 (from 32 to 129: Nachman et al., 2022; College Autism Network). Given the proliferation of these programs, it is somewhat surprising that so few young adults in the current study were participating in either of these types of programs.

Further research is needed to understand whether the low rates of participation in disability or autism-specific PSE programs are unique to this sample and, if they are not, the facilitators and barriers to participation. Low rates of participation may reflect the age range of the present subsamples– particularly Subsample 4 which included adults up to 35 years of age, many of whom would be out of the targeted age range for PSE programs– or it may suggest that these programs remain relatively inaccessible for many with autism. Inaccessibility may be due to limited capacity of programs to serve the number of students who would like to participate, or it could be due to family financial constraints. Inaccessibility could also be influenced by geographic constraints of these programs, which might be a particularly salient barrier among autistic individuals. Challenges in daily living skills are common among autistic adults (even for those with higher IQ scores; Duncan & Bishop, 2015), which may impact their ability to participate in PSE more generally, and specifically may limit how far they are able to travel for a PSE program. It is possible that college programs for students with disabilities will continue to grow (and expand to more geographic locations), and if so, more autistic students will have access and thus these new educational codes will be useful in capturing participation in these programs.

#### Limitations and Next Steps

The original Vocational Index has demonstrated usefulness in coding vocational and educational activities, and there are important next steps that will need to be taken to better understand the additional contributions of the updated codes. First, research is needed to understand whether prediction of vocational/educational activities is different when using the original Vocational Index codes versus the updated VEI codes. Similar patterns of prediction between these two coding schemes would suggest that either is useful in research contexts when activities are aggregated across individuals (as they lead to the same conclusions), and the main contribution of the updated codes may instead be in situations where capturing individual-level data is of greatest importance, such as in service contexts or when the focus is on describing activities. Alternatively, different patterns of prediction between the original and updated codes could provide rationale for using the updated codes in research contexts.

The VEI considers whether supports are provided when coding activities, but it does not attempt to categorize the extent or level of supports. There is tremendous variability in the types and intensity of supports that adults receive in their vocational and educational activities, ranging from monthly check-ins with agency personnel to one-on-one continuous support. For studies that are interested in investigating the nuance of provided supports, additional work will be needed to categorize the wide variety of supports in a way that can be applied reliably across studies. This work could result in a supplementary guide for coding support that considers multiple dimensions, including (but not limited to) the intensity of the supports provided and whether supports are provided through paid agency staff or unpaid supporters (e.g., parents). Similarly, though the VEI considers whether activities are happening for a minimal versus more-than-minimal amount of time, it does not differentiate full-time versus part-time work. There is heterogeneity in preferences for full-time versus part-time work in the autism community (e.g., Hayward et al., 2019); further research is needed to understand preferences and implications of number of hours participating in vocational/educational activities in this group. Depending on the needs of the project, there are multiple ways that researchers could study the number of hours in conjunction with the VEI. For example, the VEI could be “customized” by weighting VEI category by number of hours, or a separate variable could be constructed that captures number of hours to be used in combination with VEI codes (for a full discussion of number of hours, see Taylor & Seltzer, 2012).

Though most of the modifications to the original vocational index were focused on PSE activities, there may need to be additional adjustments to the vocational categories as the knowledge base around work activities for autistic adults continues to evolve. We coded structured unpaid internships as separate from volunteering in the VEI. However, the evidence for when and which of these programs leads to integrated competitive employment is limited (Avellone et al., 2023). Further research on internships will continue to inform how to code these activities on the VEI. Similarly, there were work activities that remained difficult to code even with the clarifications in the updated codes. Self-employment was a common example; in these cases, it could be unclear how to draw the line between independent competitive work and supported work and even to discern whether the activities were paid. It may be that additional criteria will need to be developed to code these activities more clearly.

Finally, it will be important for future research to test the usefulness of the VEI in diverse samples. Though our combined sample was diverse in functional abilities, it was not particularly diverse in terms of racial and ethnic identity or family socio-economic status. There may be vocational and PSE activities more common in groups under-represented in our samples, that are not fully addressed in the updated codes. Recruiting samples that are enriched for racial, ethnic, and other forms of diversity will be necessary to understand how the VEI performs in these groups. The updated codes should also be tested in samples of mid-life and older adults with autism, as adults in this study were all in early adulthood.

In conclusion, the development of the VEI represents a significant step forward in capturing the evolving landscape of vocational and educational activities among autistic adults. Continued refinements will ensure that the index is applicable across varied populations and age groups, enhancing the utility of the VEI as a tool for both research and practice.

## Electronic supplementary material

Below is the link to the electronic supplementary material.

Supplementary Material 1
