# Supplementary material for: The Vocational and Educational Index: An Update to the Vocational Index to Reflect Contemporary Postsecondary Educational Options for Autistic Adults
Source: J Autism Dev Disord. 2025 Feb 22;56(7):2780–9. doi: 10.1007/s10803-025-06737-8 (PMC12353770; doi:10.1007/s10803-025-06737-8)
Supplement: Supplementary file 1 — Supplementary Material 1 [file 10803_2025_6737_MOESM1_ESM.pdf]

SUPPLEMENTAL MATERIAL

Inclusion Criteria and Recruitment Methods for Each Study from Which Participants were Drawn for the Present Analysis

Subsample 1. Subsample 1 included participants from a randomized controlled trial (RCT) of a parent advocacy intervention for youth with autism. Eligibility criteria for the RCT were as follows: (1) parent or legal guardian of a youth with autism who was 16-26 years old; (2) parent provided documentation that the youth had received an autism diagnosis from a school-based or medical professional; (3) parent lived in a state in which the study was conducted (IL, TN, WI) and was able to attend the 12-week group program at the intervention site on the day and time it was delivered; (4) youth had lifetime scores on the Social Communication Questionnaire (Rutter et al., 2003) – a parent report screener for autism – of 12 or greater, indicating a likelihood of autism (a cut-off of 12 is recommended for samples that include adults with intellectual disability; Brooks & Benson, 2013). Families were recruited through autism studies, research registries, disability agencies, school personnel, and autism support groups. Further information can be found in Taylor et al., 2023.

Subsamples 2 and 3. Subsamples 2 and 3 were recruited from the Simons Simplex Collection registry (SSC; Fischbach & Lord, 2010), the Simons Foundation Autism Research for Knowledge (SPARK) research match registry (Feliciano et al., 2018), and other research and clinical databases maintained at the investigators' institutions. All youth in these registries had been diagnosed with autism spectrum disorder (ASD). Depending on the database from which they were drawn, the ASD diagnosis was either confirmed at study entry by a clinician utilizing information from well-validated instruments such as the Autism Diagnostic Observation Schedule-2 (Lord et al., 2012), or was made by a community professional and confirmed by

parental report (Feliciano et al., 2018; Fombonne et al., 2022). The SSC, SPARK, and clinical research registries used information from their databases (collected during initial registration) to identify families that met the inclusion criteria for the study. Each registry shared the study information with eligible families via email. If families expressed interest in participating, their information was passed on to the study team, who contacted them via telephone to further explain the study and to ensure eligibility.

Eligibility criteria for the larger study from which Subsample 2 was drawn were as follows: (1) youth with autism is between the ages of 15 and 25 at the time of recruitment; (2) youth has an IQ score of 70 or lower in their respective database; and (3) a parent is able and willing to fill out an online survey. Eligibility criteria for the larger study from which Subsample 3 was drawn included: (1) youth with autism is between 15 to 26 years of age at the time of the study; (2) youth had received an autism diagnosis before the age of 18; (3) youth had a historic IQ or parent-report IQ of 70 or above in their respective database; (4) youth was able to self-report and consent independently (assent for youth under 18). To confirm that youth were able to self-report, the study team verbally administered a short series of questions that assessed their language proficiency (Bishop et al., 2017). Further information about these studies can be found in Taylor and colleagues (2024) and Libster and colleagues (in press).

Subsample 4. Subsample 4 was drawn from a longitudinal study of autistic adults who could self-report. Participants were recruited from several sources, including other autism studies, research registries, disability agencies, school personnel, email listservs, and autism support groups. Inclusion criteria for the larger study were as follows: (1) adult has an ASD diagnosis provided by an educational or health professional and can provide documentation of that diagnosis; (2) adult is between the ages of 18 and 45; (3) adult has exited high school; and

(4) adult is capable of self-report. Similar to Subsample 3, ability to self-report was assessed through a series of language proficiency questions (Bishop et al., 2017). One informant (most often a parent) participated along with each autistic adult whenever feasible. Additional information about the larger study from which Subsample 4 was drawn can be found in Mueller and colleagues (under review).

Table S1

Category Percentages of Vocational Index Codes for Each Subsample Included in the Present Analyses

Percent of subsample with each code

Code	Subsample 1 (*n* = 59)				Subsample 2 (*n* = 82)				Subsample 3 (*n* = 77)				Subsample 4 (*n* = 166)			
		VEI				VEI				VEI				VEI		
	VI	Overall	Vocation	Education	VI	Overall	Vocation	Education	VI	Overall	Vocation	Education	VI	Overall	Vocation	Education
9	35.6%	35.6%	22.0%	18.6%	6.1%	6.1%	4.9%	1.2%	64.9%	62.3%	31.2%	40.3%	59.6%	60.2%	48.2%	21.1%
8	5.1%	5.1%	6.8%	0	1.2%	1.2%	0	1.2%	6.5%	7.8%	11.7%	6.5%	10.2%	10.2%	7.8%	7.2%
7	6.8%	8.5%	6.8%	3.4%	4.9%	4.9%	4.9%	0	1.3%	2.6%	1.3%	1.3%	6.6%	7.2%	7.8%	0.6%
6	1.7%	1.7%	1.7%	0	6.1%	6.1%	6.1%	0	2.6%	2.6%	3.9%	0	0.6%	1.2%	1.2%	0
5	3.4%	3.4%	5.1%	0	3.7%	6.1%	4.9%	2.4%	0	0	0	1.3%	1.2%	1.2%	0.6%	1.2%
4	8.5%	10.2%	8.5%	3.4%	29.3%	29.3%	28.0%	1.2%	0	0	0	0	2.4%	3.0%	2.4%	1.2%
3	3.4%	3.4%	3.4%	0	4.9%	4.9%	4.9%	0	0	0	0	0	3.6%	3.6%	3.6%	0
2	11.9%	8.5%	8.5%	6.8%	9.8%	7.3%	8.5%	0	1.3%	1.3%	6.5%	2.6%	3.6%	1.2%	3.6%	1.8%
1	23.7%	23.7%	37.3%	67.8%	34.1%	34.1%	37.8%	93.9%	23.4%	23.4%	45.5%	48.1%	12.0%	12.0%	24.7%	66.9%

Note. VI = Vocational Index (i.e., original codes). VEI = Vocational and Educational Index (i.e., updated codes). The Overall Code is the higher of the updated vocation or education code for each person.

Administration Procedures

Information needed to code vocational and educational activities can be generated a number of different ways and thus there is no specific protocol that is necessary to follow to implement the coding rubric. In this supplemental material, we provide some additional guidance on both data collection and coding that can be helpful for those who would like to generate VEI codes, as well as example questions that can be used to gather the information. The information required for coding an individual's vocational and educational activities can be gathered flexibly through various means, such as a survey or a semi-structured interview. Interviews/surveys can be administered to various groups of respondents, including adults with autism, family members of adults with autism, friends of adults with autism, or disability professionals familiar with the activities of the adult. Though the specific questions can differ based on the needs of the study, to generate VEI codes, the following aspects of activities should be queried: the activity's level of community integration; supports received; number of hours engaged in an average week; whether vocational activities are paid; and the expected outcome of a PSE program (i.e., certificate, degree, none of these).

Additional Coding Conventions

In coding vocational and educational activities for the present analysis, we developed coding conventions that may be useful in other contexts. These relate to: (1) determining a code when there are multiple vocational and/or multiple educational activities, (2) the timing of the interview, (3) coding vocational training; (4) considering non-traditional work formats; and (5) coding previous vocational/educational activities.

Multiple activities. It was relatively common to encounter cases in which adults had two or more activities that would be coded within the same category, such as two part-time jobs in

the community without supports. Sometimes the amount of time spent in each of these activities was ten hours or less, but the total number of hours of activity in that category was above ten. In these cases, we recommend adding the hours of activity within a category to decide whether to code the activity as minimal hours. For example, if an adult had one job in the community without support for 6 hours a week and another community job without support for 8 hours a week, they would get a code of “9” for the vocational dimension even though neither job, on its own, was over ten hours a week.

Different considerations come into play if a person has two (or more) activities that fall into different categories within the vocational or educational dimension. In these cases, we recommend that the vocational code reflect the highest code for activities within that dimension. Consider the example of an adult with two jobs: job 1 is in the community with supports for 15 hours a week at a doctor’s office, and job 2 is in the community without support at a movie theater for 6 hours a week. The office job would get a code of “7” and the movie theater would get a code of “8:” The overall vocational dimension code would be the higher of the two, or an “8.”

Interview timing. Though the VEI is generally intended to capture current postsecondary vocational/educational activities, situations sometimes arise in which individuals have upcoming plans for a particular vocational or educational activity (e.g., “I will be starting a job next week.” or “I will be going to college in the fall.”). When encountering these situations, it may be helpful to decide a priori on an interval of time around the data collection date, and code vocational/educational activities that the adult participates in within that interval (e.g., 2 weeks before or after the information on vocational/educational activities is collected). The length of the interval will be dependent upon the overall goal of the specific research project, as well as the

data collection timepoint(s). If data collection is repeated (such as for intervention and longitudinal studies) and relatively frequent, the interval of time in which one might consider an activity “current” will likely be shorter than if the data is collected at only one time point or at less frequent intervals.

Vocational training. In coding vocational and educational activities, we encountered individuals who were engaged in vocational training across many different venues. Vocational training happened within the context of PSE programs for students with disabilities, but it also occurred through more traditional job training or job preparation programs. Given the variety of places where vocational training can be obtained, one challenge is deciding whether to code vocational training as a vocational activity or as an educational activity. To make this distinction, in coding for the present study, we relied on thinkcollege.net, which is a regularly updated catalogue of PSE programs for students with disabilities. If the vocational training program described by participants was listed in thinkcollege.net, it was coded under the educational dimension of the VEI. If it was not, it was coded under the vocational dimension.

Non-traditional work formats. Non-traditional work formats are becoming increasingly common among employees with autism or other disabilities, due (at least in part) to the rise of the gig economy as well as initiatives in some states in the United States to fund entrepreneurial ventures as part of their disability employment services. Many adults in our studies engaged in these types of employment activities, and we found that the VEI was able to characterize their work. Similar to more traditional employment activities, for non-traditional work formats we considered: a) whether the adult was receiving income from the activities; b) whether they were performing the activities with support above and beyond what might be available in the general

population; and c) whether the activities were segregated from the general population, integrated, or a combination thereof.

Coding previous vocational/educational activities. The VEI is typically used to code current vocational/educational activities, but the interview used to gather information on activities (included in this supplemental material) can be modified to also capture history of activities over a defined period (e.g., over the past five years; since high school exit). After gathering information on history of activities, VEI codes can be applied to each activity, and then researchers can use these codes in whatever way best supports their research question of interest. For example, using the interview in this way could be helpful in determining whether autistic adults participated in community work after high school exit, or in investigating work activities after finishing a PSE program.

EXAMPLE VOCATIONAL AND EDUCATIONAL INTERVIEW

Version Date: 7/20/2024

General Activities

For this interview, I'm going to ask you about academic programs, employment, and volunteering activities that you are currently participating in.

First, I'd like to get an idea of how you usually spend your time. Let's talk about the activities you participate in during a typical week.

1. **What does a typical week look like for you? How are you spending your time?**

   NOTE: This description can include activities like school programs, work, and volunteering.
   We will NOT include recreational or social activities in this measure.

2. **Please tell me if you are CURRENTLY involved in any of the following activities:**

   NOTE: Select all that apply. Hopefully notes from Q1 will inform this list, but it may also be helpful to ask about certain activities if they did not bring them up before.

   - ❑ (1) Postsecondary education program
     (e.g. traditional college, trade school, an academic program for individuals with IDD)
   - ❑ (2) Vocational training program
   - ❑ (3) Adult day program
   - ❑ (4) Sheltered workshop
   - ❑ (5) Working for pay with support
     (i.e. supported or customized employment)
   - ❑ (6) Working for pay independently (i.e. competitive employment)
   - ❑ (7) Volunteering in the community
   - ❑ (8) Other/not sure
     (a) Please specify:

____

____

Postsecondary Educational Programs

Now we'll talk about **school programs** that you may be attending currently….

3. **Are you currently enrolled in an academic program at a vocational school, community college, college, or university?**

    a. No *SKIP TO Q6 P.4 (section labeled vocational activities).*

    b. Yes

Please tell me about those schools or programs.

(can repeat page 3 if more than two schools/programs)

Use the following codes to complete the tables on the next pages:

**Type of Program**	**Type of Supports** *(choose all that apply)*
❑ (1) Postsecondary education program for people with disabilities; may be attending tech/vocational/college class, but not currently pursuing a regular degree<br>❑ (2) Taking classes at a community college, college, or university; not degree-seeking<br>❑ (3) Working towards a certificate at a vocational, business, or technical school; not degree seeking<br>❑ (4) At a 2-year college or community college, working towards an associate's degree<br>❑ (5) At a 4-year college or university, working towards a bachelor's degree<br>❑ (6) Graduate or Professional degree<br>❑ (7) Other/not sure	❑ (0) no supports<br>❑ (1) extra time on tests<br>❑ (2) note takers<br>❑ (3) assistive technology in class<br>❑ (4) accessible class materials (i.e., alternate format exams, audio textbooks)<br>❑ (5) tutoring of social aid from similar-aged peer<br>❑ (6) aid from school or program staff<br>❑ (7) other type of support (describe)

3.	PSE Program 1	PSE Program 2
**Name & location of program**		
**General description of program**		
**Disability-specific program or program with autism-specific supports?**	❑ Yes<br>❑ No<br><br>If yes, describe:<br>____________________<br>____________________	❑ Yes<br>❑ No<br><br>If yes, describe:<br>____________________<br>____________________
**Type of degree or certificate** (e.g., B.A., technical certificate)		
**Code for Type** (see code)		
**Area of Study** (or classes taken)		
**Avg. # of classes**		
**Avg. total time in and out of class**		
**Enrollment**	❑ Full-time<br>❑ Part-time	❑ Full-time<br>❑ Part-time
**Start Date** (Month/Year)		
**Supports** (see codes)	❑ Yes Types of supports:<br>____________________<br>____________________<br>____________________<br><br>❑ No Why not?<br>❑ Supports not available<br>❑ Supports available but youth not using them<br>❑ Other<br>❑ N/A – not needed	❑ Yes Types of supports:<br>____________________<br>____________________<br>____________________<br><br>❑ No Why not?<br>❑ Supports not available<br>❑ Supports available but youth not using them<br>❑ Other<br>❑ N/A – not needed
**Additional Comments**		

Postsecondary Vocational Activities

Now we would like to know about your employment, vocational training, and volunteering activities that you may be participating in currently.

3. **Are you currently participating in any employment, vocational, or volunteering activities?**
   a. No *END INTERVIEW*
   b. Yes

Please tell me about those jobs or programs, beginning with the one where you spend the most time.<br><br>*(can repeat page 5 if more than two jobs or programs)*

Use the following codes to complete the tables on the next pages:

Type of Activity
❑ (1) Volunteering in the community<br>❑ (2) Vocational training program<br>❑ (3) Sheltered/segregated vocational activities (e.g. adult day program, sheltered workshop)<br>❑ (4) Some segregated AND some integrated vocational activities (e.g. both sheltered workshop and supported employment)<br>❑ (5) Working for pay with support (i.e. supported employment)<br>❑ (6) Working for pay independently (i.e. competitive employment)<br>❑ (7) Other/not sure

7.	Vocational Activity 1	Vocational Activity 2
**Name of Employer**		
**Job/activity title and brief description**		
**Day-to-Day activities and responsibilities**		
**Supports**<br>**(i.e., job training, job coach, transportation provided by voc rehab, postsecondary program, family member)**	❑ Yes, supported<br>❑ No, competitive<br><br>Describe supports and provider:<br>______________________________<br>______________________________	❑ Yes, supported<br>❑ No, competitive<br><br>Describe supports and provider:<br>______________________________<br>______________________________
**Paid**<br>**(do you receive a salary? how much per hour, week, month or year?)**	❑ Yes<br>❑ No<br><br>Describe amount of pay:<br>______________________________<br>______________________________	❑ Yes<br>❑ No<br><br>Describe amount of pay:<br>______________________________<br>______________________________
**Type of Activity**<br>**(see code)**		
**How did you find this activity?**<br>**(i.e., job service, school, family, friends, or on your own)**		
**Hours per week**	__________ hours per week	__________ hours per week
**Start Date**<br>**(Month/Year)**		
**Additional Comments**		
